# Viral metagenomic analysis of bushpigs (*Potamochoerus larvatus*) in Uganda identifies novel variants of Porcine parvovirus 4 and Torque teno sus virus 1 and 2

**DOI:** 10.1186/1743-422X-9-192

**Published:** 2012-09-11

**Authors:** Anne-Lie Blomström, Karl Ståhl, Charles Masembe, Edward Okoth, Ademun Rose Okurut, Patrick Atmnedi, Stephen Kemp, Richard Bishop, Sándor Belák, Mikael Berg

**Affiliations:** 1 Department of Biomedical Sciences and Veterinary Public Health, Section of Virology, Swedish University of Agricultural Sciences, Uppsala, Sweden; 2 Department of Virology, Immunobiology and Parasitology (VIP), National Veterinary Institute (SVA), Uppsala, Sweden; 3 Department of Biology, Makerere University, College of Natural Sciences. School of Biological Sciences, Box 7026, Kampala, Uganda; 4 International Livestock Research Institute (ILRI), Nairobi, Kenya; 5 National Animal Disease Diagnostics and Epidemiology Centre (NADDEC), Ministry of Agriculture Animal Industry and Fisheries, Entebbe, Uganda; 6 Uganda Wildlife Authority, Kampala, Uganda

## Abstract

**Background:**

As a result of rapidly growing human populations, intensification of livestock production and increasing exploitation of wildlife habitats for animal agriculture, the interface between wildlife, livestock and humans is expanding, with potential impacts on both domestic animal and human health. Wild animals serve as reservoirs for many viruses, which may occasionally result in novel infections of domestic animals and/or the human population. Given this background, we used metagenomics to investigate the presence of viral pathogens in sera collected from bushpigs (*Potamochoerus larvatus*), a nocturnal species of wild Suid known to move between national parks and farmland, in Uganda.

**Results:**

Application of 454 pyrosequencing demonstrated the presence of Torque teno sus virus (TTSuV), porcine parvovirus 4 (PPV4), porcine endogenous retrovirus (PERV), a GB Hepatitis C–like virus, and a Sclerotinia hypovirulence-associated-like virus in sera from the bushpigs. PCR assays for each specific virus combined with Sanger sequencing revealed two TTSuV-1 variants, one TTSuV-2 variant as well as PPV4 in the serum samples and thereby confirming the findings from the 454 sequencing.

**Conclusions:**

Using a viral metagenomic approach we have made an initial analysis of viruses present in bushpig sera and demonstrated for the first time the presence of PPV4 in a wild African Suid. In addition we identified novel variants of TTSuV-1 and 2 in bushpigs.

## Background

Wild animals are carriers of a number of pathogens that have the potential to infect the human population and/or domestic animals. The intensity of contact between wildlife, livestock and human population is increasing due to a number of factors, primarily human and livestock population growth leading to encroachment onto wildlife habitat [1,2]. During recent decades a number of pathogen crossovers from wildlife to humans and livestock have occurred resulting in emerging diseases, such as SARS, Hantavirus Pulmonary syndrome 1, Nipah virus disease 1, and Hendra virus-induced diseases among others. However, it is also evident that transmission can occur both ways i.e. pathogens may spill-over to wildlife from humans and/or from livestock. One example of this is the spill-over of Canine distemper virus from domestic dogs (*Canis familiaris*) to African wild dogs (*Lycaon pictus*) in Serengeti in 1991 leading to local extinction of wild dogs in the area [2]. Apart from being carriers of novel viruses with the potential to cause disease in naïve domestic hosts, wildlife may also act as reservoirs for known viral pathogens – for example there are a number of wildlife reservoirs for foot and mouth disease virus such as African buffalo (*Syncerus caffer*), reindeer (*Rangifer tarandus*) and wild boar (*Sus scrofa*) [3]. However, information on the viral flora in wildlife is typically scarce or non-existent.

Traditional viral detection methods, such as virus isolation, are often hindered by the inability to grow virus in cell culture. The divergence of many viruses and absence of a common viral marker gene makes detection difficult using standard molecular techniques including PCR and microarray as they are frequently target-specific through the use of specific primers, probes and/or antibodies. Viral metagenomics is a sequence, and culture-independent approach that has proven to be a valuable tool for the investigation not only of diseases of unknown etiology but also of the complete viral flora of different reservoirs and vectors. By providing insights into a wide range of unknown potential pathogens and revealing novel aspects of biodiversity, metagenomics is able to detect and characterise novel pathogens [4-6].

In many rural parts of the developing world, domestic livestock are kept in free-range systems, potentially allowing contact with wild animals. In some parts of Uganda, free-range scavenging by pigs is frequent. At the same time wild species of suidae such as bushpigs (*Potamochoerus larvatus*), with a wide distribution in Eastern and Southern Africa, live and move at the interface between the national parks and the farmland where there is an opportunity for interaction and sharing of pathogens with domestic relatives. Bushpigs are considered to be possible natural reservoirs for African swine fever virus [7], but less is known about what other viruses bushpigs might carry. Therefore, to investigate whether bushpigs are carriers of known and or unknown porcine viruses we have in this study investigated the viral flora of bushpig sera.

## Results and discussion

A total of 171,466 reads were obtained from the 454-sequencing run, and after assembly 4,441 contigs were created while 32,863 reads remained unmatched (singletons). Although blastn and blastx analysis showed that a majority of the sequences were non-viral, 35 contigs and singletons sequences were identified as viral sequences (Table1). The high percentage of host genetic sequences found despite of the nuclease treatment prior to sequencing demonstrates the difficulties of completely reducing the background of the host as discussed in the review by Blomström A-L 2011 [4]. Most viral hits showed closest similarity to known pig viruses such as, Torque teno virus (TTSuV) and porcine parvovirus 4 (PPV4). By design of primers based on the sequences obtained from the 454-sequencing run, PCR assays were established to verify the presence of these viruses. These PCR assays confirm the presence of TTSuV1, TTSuV2, PPV4, and the porcine endogenous retrovirus (PERV). The GB Hepatitis C-like, and the Sclerotinia hypovirulence associated like virus, on the other hand, were not detected by the PCR approach, possibly due to the low concentration of these viruses in the samples as only one sequencing read was found for each among the total 171,466 reads obtained from the 454-sequencing run. Also it is possible that in some cases sequencing errors led to primer mismatches.

**Table 1:** Viral hits of the 454 reads after assembly and blastn/x searches

**Virus hit**	**Blastn identity**	**Blastx identity**
TTSuV-1	75 - 83%	48 - 75%
TTSuV-2	68 - 85%	40 - 86%
PPV4	No hit - 72%	40 - 76%
GB Hepatitis C virus	No hit	33%
Sclerotinia hypovirulence associated virus	78%	71%
Porcine endogenous retrovirus	80 - 95%	86 - 89%

The table shows the sequence similarity that the 454 reads, identified as viral sequences through blastn and blastx searches, had to best available hit in GenBank.

### Parvovirus

Parvoviruses are small single-stranded linear DNA viruses with a genome of approximately 5000 nucleotides, which have been found in a number of species such as human, swine, cattle and gorilla [8]. In swine, porcine parvovirus (PPV) is a known agent that causes reproductive failure [9]. However, in recent years a number of new parvoviruses – porcine hokovirus (PHoV) [10], porcine bocavirus (PBoV) [11] and porcine parvovirus 4 (PPV4) [12] - have been discovered in pigs, with their potential involvement in disease currently unknown.

**Figure 1:**
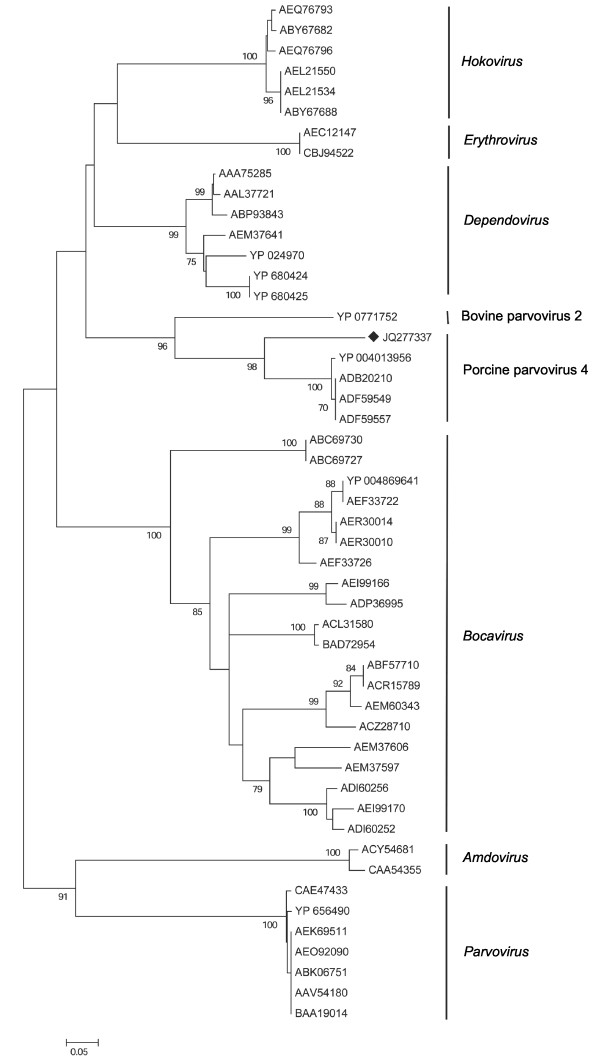
**Neighbour-joining phylogenetic tree of Parvovirus.** The Neighbour-joining tree shows the phylogenetic relationship between the bushpig PPV4 sequence (84 amino acid long) and 49 sequences available from GenBank. The bushpig PPV4 is indicated with ♦.

The parvovirus sequences discovered in the investigated bushpigs showed closest similarity to PPV4. Porcine parvovirus 4 was originally discovered in 2010 in samples collected from pigs in North Carolina in 2005 after an outbreak of acute-onset of disease with high mortality [12]. Subsequently, PPV4 was reported in pigs in China where 1.84% of the investigated pigs were PPV4 positive [13]. The genome of parvoviruses consists of two major open reading frames (ORF) encoding the non-structural and the capsid proteins. However, the genomes of PPV4 as well as of PBoV contain an additional third ORF [12]. The parvovirus sequences obtained from this study could be found in the capsid and in the non-structural ORF. All reads classified as parvovirus gave significant similarity to PPV4 through Blastx searches. A PCR with primers designed to amplify PPV4-like sequences from the original extracted genetic material showed the presence of this virus in one of the three bushpig sera. Sequencing of the PCR product (GenBank accession number: JQ277337) confirmed correct amplification and sequence analysis showed that at protein level (84 amino acids), the product displayed a 75.9-77.1% sequence similarity to the PPV4 sequences available in GenBank. In addition, the phylogenetic tree generated from these data (Figure1) grouped the sequence with PPV4 when analysed together with sequences from all the different parvovirus genera (*ParvovirusErythrovirusDependovirusAmdovirus* and *Bocavirus*) and PPV4. However, the tree also confirms the divergence of the bushpig PPV4 from the other PPV4.

PPV4 has previously been reported in domestic pigs only in USA and China [12,13]. However with this study we show the presence of a PPV4 variant in a wild suid in Uganda.

### Torque teno sus virus

Torque teno virus was discovered 1997 in a serum sample from a patient with posttransfusion hepatitis of unknown etiology using representational difference analysis [14]. Since then the virus has been detected and characterised in a number of species such as primates, cats, dogs and pigs [15], but the role of these viruses in disease development is still controversial. These viruses have small (approximately 2.8 kb) circular DNA genomes. Torque teno virus in pigs is divided into two different species, Torque teno sus virus 1 and 2, and prevalence studies have shown that TTSuV is widely spread in pig populations across the world [16,17]. In a previous study [18], we have found that 51.6% of a sample population of domestic pigs in Uganda were carrying one or both these TTSuV variants. Although, most studies have targeted domestic pigs, TTSuV have also been found in wild boar in Europe [19].

As shown in Table1, our data indicated the presence of both TTSuV-1 and 2 in the investigated serum samples. Both the TTSuV-1 and 2 sequence reads were located in the major open reading frame (ORF1) and all reads showed significant similarity to the respective virus in both blastn and blastx analyses.

**Figure 2:**
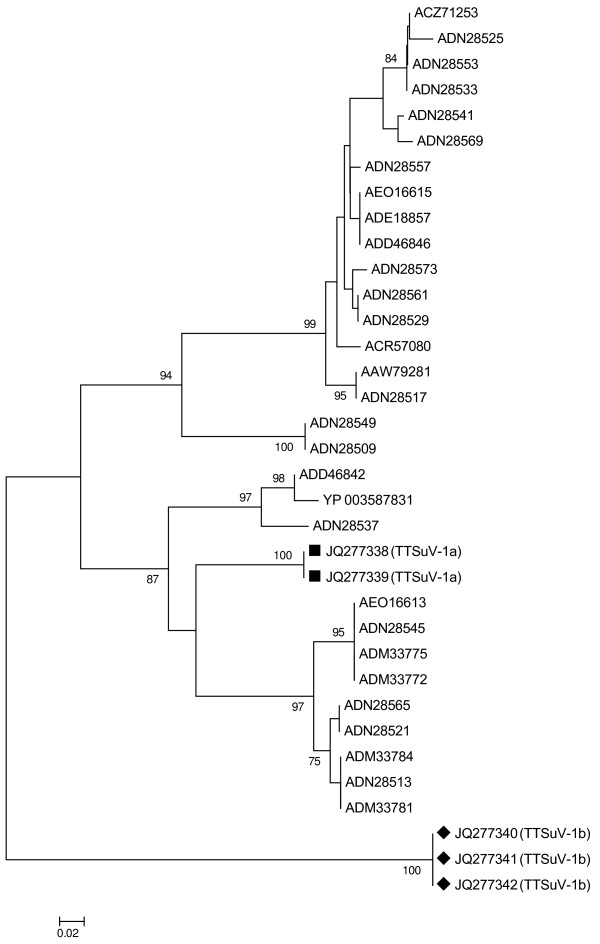
**Neighbour-joining phylogenetic tree of TTSuV-1.** The Neighbour-joining tree shows the phylogenetic relationship between the five bushpig TTSuV-1 sequences (66 amino acid long) and 30 sequences available in GenBank. The bushpig TTSuV-1a is indicated with ■ and TTSuV-1b with ♦.

Two of the TTSuV-1 sequence reads partially overlapped and the sequence analysis indicated a significant variation between the two and therefore two different TTSuV-1 PCR assays were designed. The results from the specific PCR assays showed that one of the TTSuV-1 variants (here named TTSuV-1a) could be found in two of the three bushpig sera while the other one (here named TTSuV-1b) was found in all three. The sequence analysis of the PCR amplified TTSuV-1 products (GenBank accession number JQ277338 - 42) showed a sequence similarity between TTSuV-1a and b on protein level at 53–54.5% in the analysed 66 amino acid region. Compared to sequences from other studies, TTSuV-1a showed a 60.6-84.8% protein sequence similarity while TTSuV-1b was more divergent (50-56% similarity). These protein sequence identity values were similar to those seen when comparing the sequences retrieved from the GenBank with each other (59-100% similarity). The phylogenetic tree (Figure2) also indicated that TTSuV-1b was more divergent than the other sequences. Sequences from different parts of the world such as China, Germany, Spain, US etc. was used in the phylogenetic study however no clear geographical clustering was seen.

**Figure 3:**
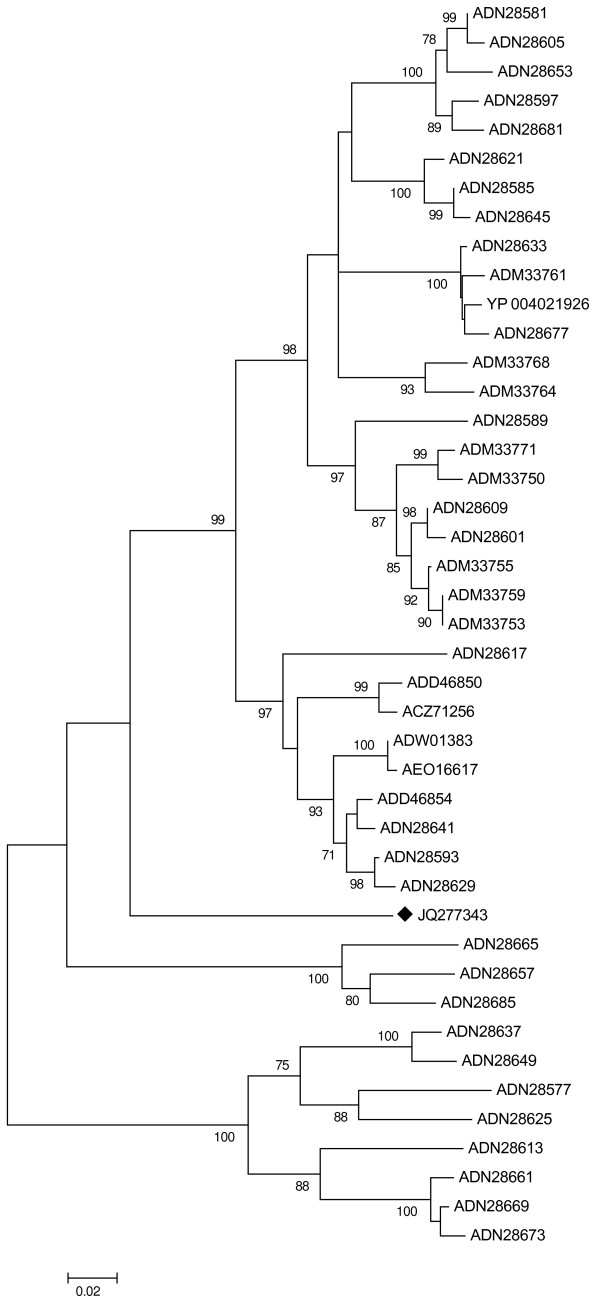
**Neighbour-joining phylogenetic tree of TTSuV-2.** The Neighbour-joining tree shows the phylogenetic relationship between the bushpig TTSuV-2 sequence (313 amino acid long) and 42 sequences available in GenBank. The bushpig TTSuV-2 is indicated with ♦.

TTSuV-2 was confirmed in one of the bushpig sera and the amplified product GenBank accession number JQ277343) showed a protein sequence identity of 66.6-79.4% to the other TTSuV-2 sequences used in the phylogenetic study using a 313 amino acid region. This sequence identity was in the range of the similarity seen between the different TTSuV-2 sequences from other studies used for comparison (64.2-100%). In the phylogenetic study (Figure3) the sequenced TTSuV-2 grouped with the other TTSuVs but in its own clade.

TTSuV-1 and 2 have previously, as mentioned, been detected both in domestic pigs across the world [16-18] and wild suidae in Europe [19] and now for the first time in bushpigs on the African continent. Studies on the genetic variability of TTSuV-1 and 2 have shown a higher genetic diversity in the coding regions compared to the untranslated region [20]. The sequence analysis of both the bushpig-derived TTSuV and those from GenBank shows a high genetic variability among the different TTSuV-1 and TTSuV-2 and also shows the co-infection of two different TTSuV-1 variants and one TTSuV-2.

### Retrovirus

Endogenous retroviruses are integrated in the host genome and all vertebrates investigated have been shown to carry retroviral sequences. It is estimated that up to 10% of animal genomes are retroviral elements [21]. Also the bushpig genome has been investigated and confirmed to contain PERV [22-24].

By running the specific PERV PCR on both DNA and on DNase treated RNA we could as expected see that all the bushpigs had both integrated proviral DNA and expressed PERV RNA, indicating active viral transcription and replication. The sequenced PERV products (GenBank accession number JX566717-719) showed a high similarity (85 - 89%) to those available in GenBank.

## Conclusions

Through investigating sera collected from bushpigs in Uganda by viral metagenomics, we have for the first time showed the presence of PPV4 in a wild Suid on the African continent. The region of PPV4 investigated indicates a sequence divergence relative to previously described PPV4. In addition, novel TTSuV-1 and 2 variants were found. Further sequence analysis and prevalence studies can be used to define the genetic relationships of these viruses and their distribution in both domestic pigs and in wildlife.

## Methods

### Samples

The sera from three bushpigs collected from Lake Mburo National Park, Uganda in March 2010, as part of a research project on African swine fever epidemiology were used in this study. The animals were captured using game capture nets (50x3m, 150 mm square mesh, 3.5 mm nylon braid khaki, ALNET Ltd, South Africa) with assistance from Uganda Wildlife Authority (UWA) staff, and sedated with zolazepam and tiletamine (Zoletil forte vet 50 mg/ml + 50 mg/ml, Virbac Laboratories, France) before blood sampling from the saphenous vein. The Ministry of Agriculture Animal Industry and Fisheries and Uganda Wildlife Authority, together with Makerere University are mandated to carry out animal disease investigations in livestock and wildlife in the country. This is done by veterinarians who handle the animals under internationally recognized guidelines.

### Sample preparation, nucleic acid extraction and random PCR

Fifty microliters of serum was aliquoted for the RNA and DNA extraction respectively. One hundred and fifty microliter of 1x DNase buffer (Roche, Mannheim, Germany) was added to each aliquot of serum after which the sample was treated with nucleases - 100 U DNase (Roche, Mannheim, Germany) and 2 μg RNase (Invitrogen, Carlsbad, CA, USA) for two hours at 37°C in order to degrade the host nucleic acid. Trizol was added to one of the two aliquots and RNA was extracted using a combination of Trizol and Qiagen RNAeasy kit. DNA was extracted using the Qiagen DNAeasy mini extraction kit according to the manufacturer’s instructions and eluted in 50 μl elution buffer (EB). The DNA and RNA were amplified by random PCR as described earlier [25]. Before sequencing, the primers were cleaved using EcoRV (NEB, Ipswich, MA, USA) and the cleaved product was purified using the Qiagen PCR purification kit (Qiagen, Hilden, Germany) and eluted in 30 μl EB.

### High-throughput sequencing and data analysis

The purified product was sequenced on 1/8^th^ of a pico titre plate using the 454 technology by Roche at Inqaba Biotech (South Africa). The sequences were analysed through quality check and removal of very short sequences before being assembled using CLC genomic workbench v4.6 (http://www.clcbio.com/index.php). Blastn and blastx searches were performed through the Camera 2.0 portal [26,27] and searches through NCBI (http://www.ncbi.nlm.nih.gov/blast/Blast.cgi). The viral blast hits with an e-value of 10^-4^ or lower were further analysed.

### Confirmation PCRs, sequencing and phylogenetic studies

PCR primers were designed based on the reads from the 454-sequencing run (Table2) and PCR assays were set up with the aim to look for each individual virus in each bushpig sera. For the RNA viruses cDNA synthesis was performed prior to PCR using Superscript III (Invitrogen, Carlsbad, CA, USA) and random primers according to the manufacturer’s instructions. The PERV RNA was treated with DNase prior to the cDNA synthesis and both a “+” and a “–“ RT cDNA synthesis reaction was performed. The PCR using each specific primer pair (Table1) was performed according to the following procedure: 1x PCR buffer, 2.5 mM MgCl_2_, 1.0 mM dNTP, 0.4 μM forward primer and reverse primer each, and 1.25 U AmpliTaq Gold DNA polymerase (Applied Biosystems, Foster City, CA, USA). For each reaction, two μl DNA or cDNA from each respective bushpig was used. The amplification was performed with the following reaction conditions: a 12 minute enzyme activation step at 95**°**C followed by 39 cycles of 95**°**C for 30 seconds, 58**°**C for 30 seconds and 72**°**C for 90 seconds, finishing with one cycle for 10 minutes at 72**°**C. The PCR products were visualized on a 1.5% agarose gel. The PCR-positive products were purified using the QIAquick PCR purification kit (Qiagen, Hilden, Germany) according to the manufacturer’s instructions and eluted in 25 μl EB. The purified products were sequenced with standard Sanger sequencing using Big Dye Termination kit (Applied Biosystems, Foster City, CA, USA) according to the manufacturer’s instructions. The obtained chromatograms were edited using SeqMan (Lasergene 9, DNASTAR Inc., Madison, USA). Sequence identity plots were performed using the Bioedit software [28]. ClustalW as well as the phylogenetic analyses were carried out using Mega 5 [29]. The phylogenetic trees were constructed using the Neighbour-joining algorithm with p-distances and with a bootstrap value of 1000.

**Table 2:** Primers for verification

**Primer**	**Sequence 5’- 3’**
TTSuV-1a_F	TCC CAG CAG AAG ATG TAG TC
TTSuV-1a_R	GGA TGG TGG CCT CTA CTA C
TTSuV-1b_F	GCA GCA TAA CGC TAG GCT G
TTSuV-1b_R	AGA GGA AAT GGG CTA CCT G
PPV4_F	CTC TGA TAA TGT ATT ACT GGT C
PPV4_R	AAG AAA GAT CCT TCT GTT ACA
GB Hepatitis C virus_F	CTG CCT CAA CGT TGA GGC AG
GB Hepatitis C virus_R	ACG ACG TAG CAG TGG TAG AT
Sclerotinia hypovirulence associated virus_F	CGC ATC ACG GTC AAG TTT GA
Sclerotinia hypovirulence associated virus_R	TTA CAT TGC TTC GTC GAC TTC
PERV_F	ATG GGA GCT GGG TCC AAT C
PERV_R	CCT TAC GTT TGA CTC TCG AC

Primers used for the detection and sequencing of the found viruses.

## Competing interests

The authors declare that they have no competing interests.

## Authors’ contributions

ALB has performed the laboratory experiments, contributed to the study design, data analysis, manuscript draft and final manuscript preparation. KS and CM coordinated the field work and contributed to study design, data analysis and final manuscript preparation. EO and PA coordinated the bushpig capture and contributed to the final manuscript preparation. ARO, SK, RB and SB contributed to the final manuscript preparation. MB contributed to study design, data analysis and final manuscript preparation. All authors have read and approved the final manuscript.
